# Supplementary figures and images for: Identification of β2 microglobulin, the product of B2M gene, as a Host Factor for Vaccinia Virus Infection by Genome-Wide CRISPR genetic screens
Source: PLoS Pathog. 2022 Dec 27;18(12):e1010800. doi: 10.1371/journal.ppat.1010800 (PMC9829182; doi:10.1371/journal.ppat.1010800)

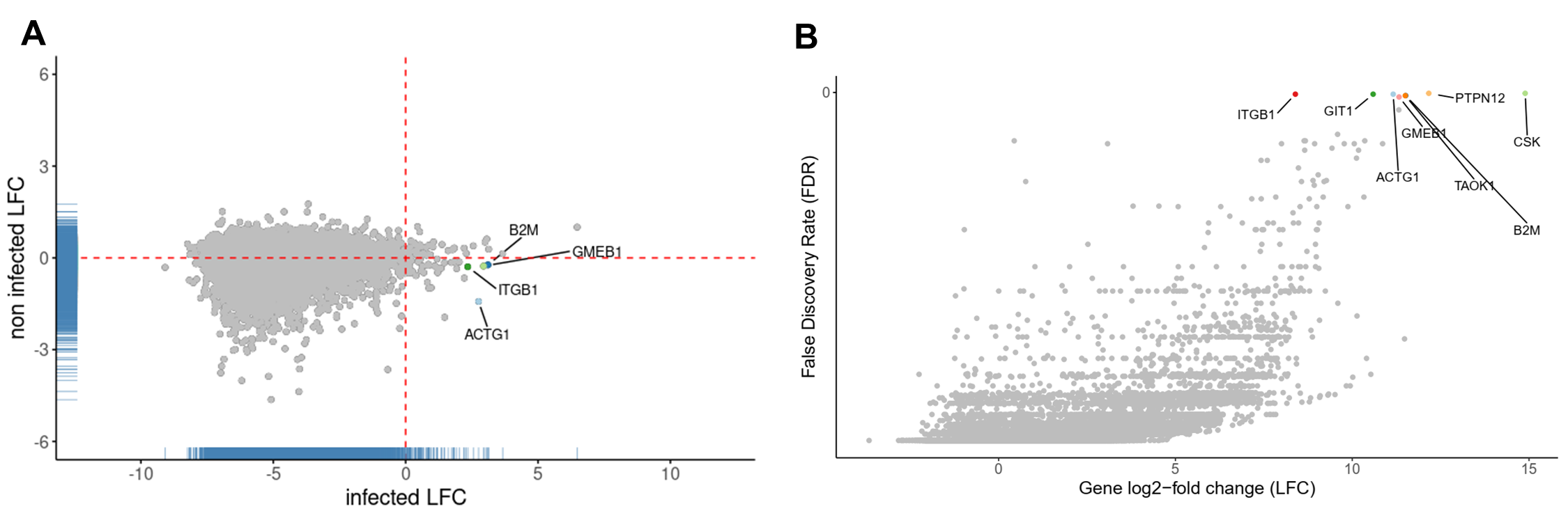

Supplement: S1 Fig — A representative hit analysis for one of the 27 screen experiments analyzed with MaGeCK (exp 1_1). A) Hits with FDR < 0.05, and LFC > 0 for infected experiments or LFC < 0 for uninfected are labelled. Best hits are those who have the greatest LFC in infected control and the lowest LFC in control experiment (uninfected). B) Volcano-like plot representing each gene KO according to their FDR (y axis) and LFC (x axis). (TIF) [file ppat.1010800.s001.tif]

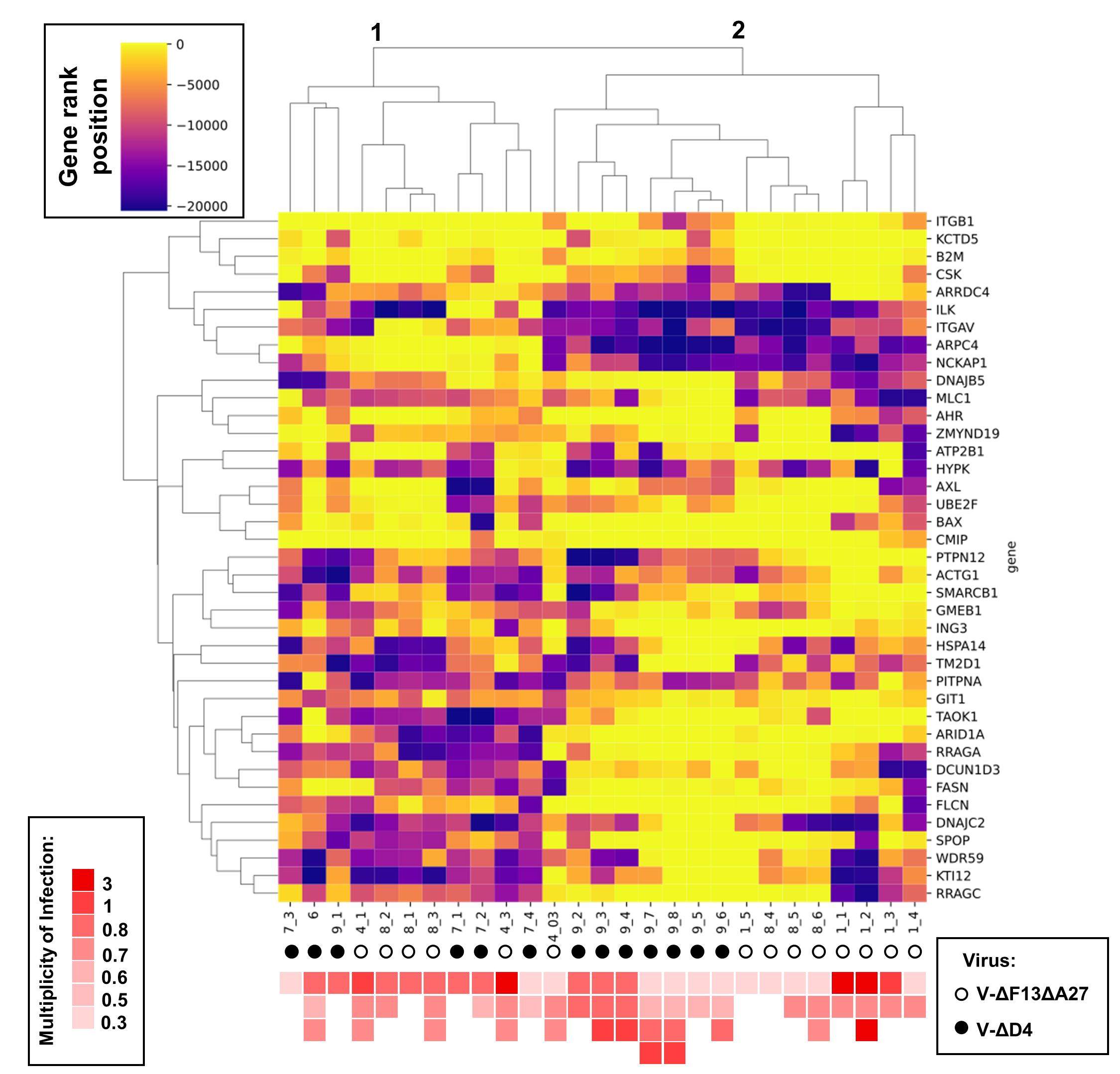

Supplement: S2 Fig — A clustering algorithm (Pearson correlation) was used to analyze the screen hits and experiments. Data was the gene rank considering the final hits list. Different rounds of reinfection and the different m.o.i. applied are indicated as red boxes. The VV mutant used in each experiment is indicated as black or white circles for V-ΔA27ΔF13 and V-ΔD4L, respectively. (TIF) [file ppat.1010800.s002.tif]

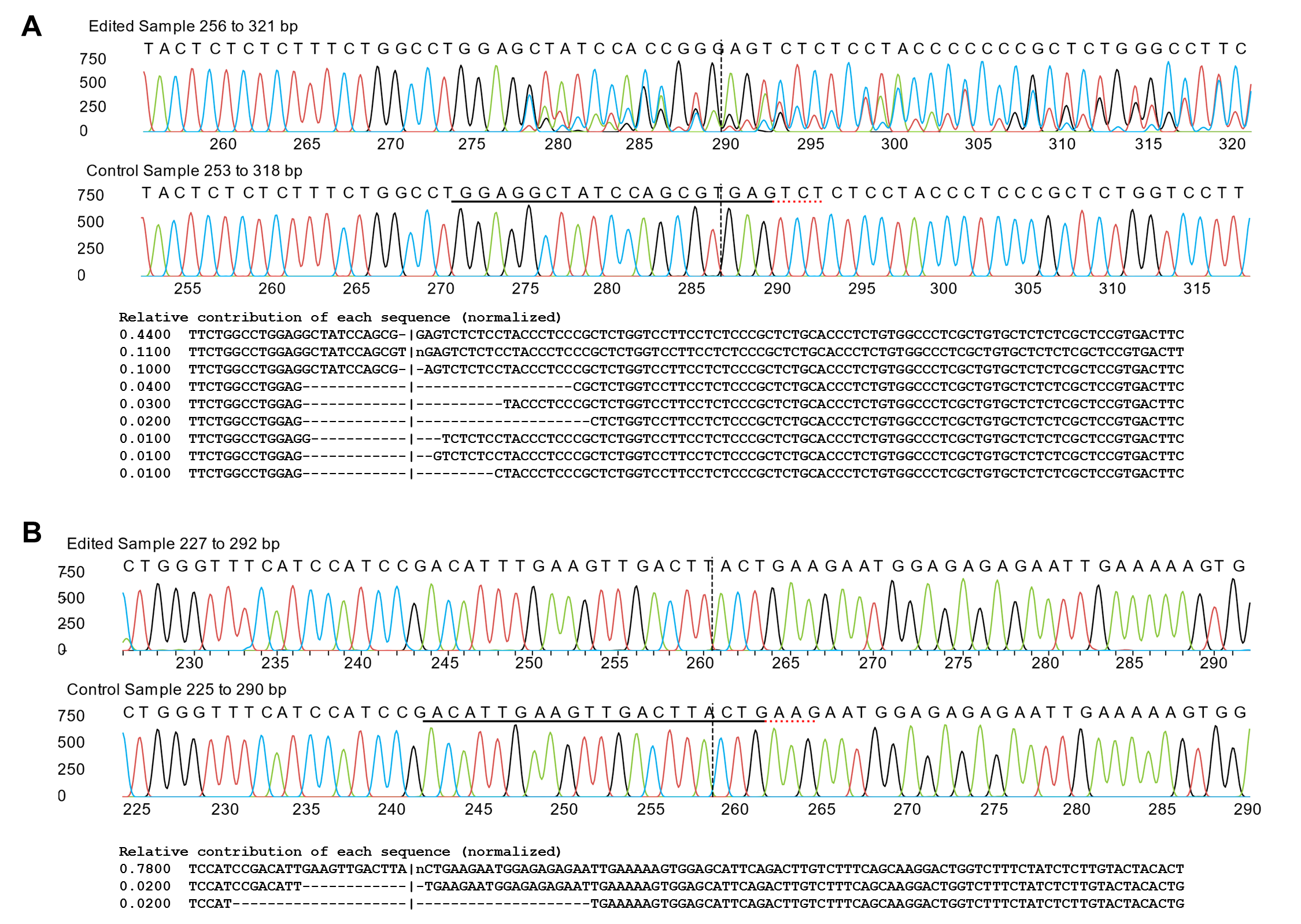

Supplement: S3 Fig — Sanger sequencing of the CRISPR/Cas9-targeted regions of both HeLa B2M KO clones compared to WT (HeLa), followed by Synthego ICE software analysis. Resulting INDEL-mutations disrupt B2M ORF at genomic level. (TIF) [file ppat.1010800.s003.tif]

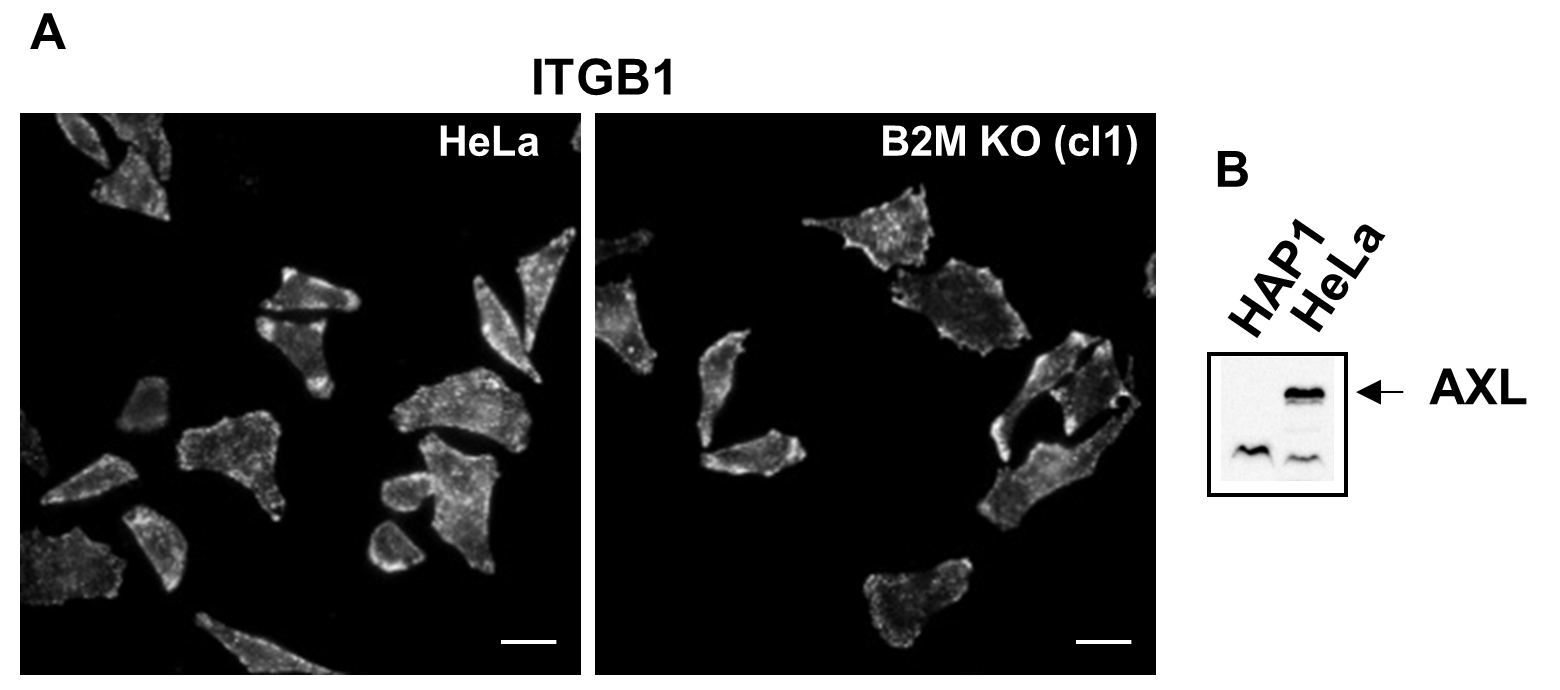

Supplement: S4 Fig — A) Immunofluorescence images of HeLa and HeLa B2M KO cells stained with anti-ITGB1 antibody. Scale bars: 20 μm. B) Western blot of whole cell lysates of HAP1 and HeLa cells, showing HAP1 cells do not express AXL protein. (TIF) [file ppat.1010800.s004.tif]

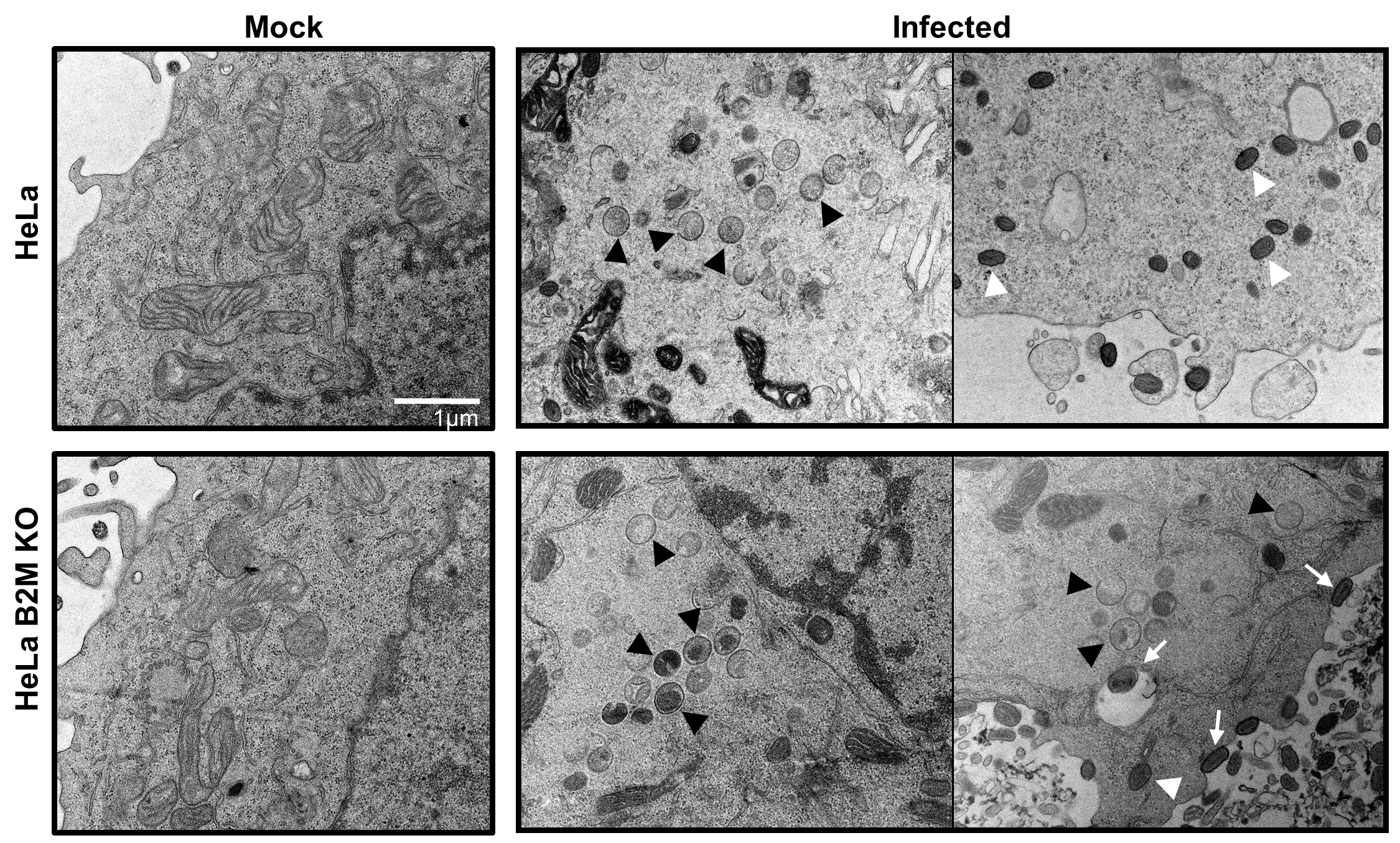

Supplement: S5 Fig — Cells infected for 18h were fixed and processed for electron microscopy. Note the presence of Immature virus particles (black arrowheads), Mature virus (white arrowheads) or enveloped virus (white arrows). (TIF) [file ppat.1010800.s005.tif]

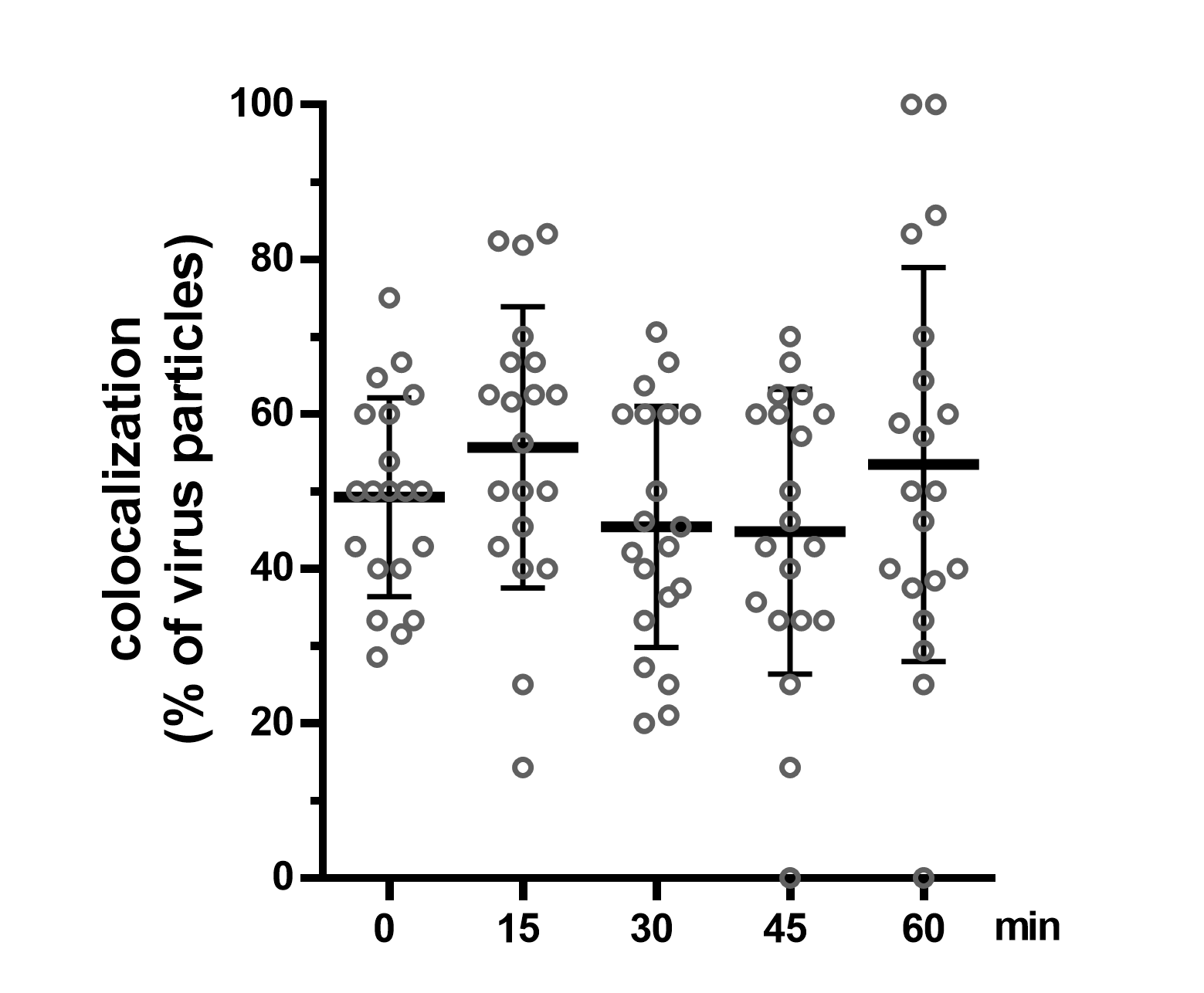

Supplement: S6 Fig — HeLa cells were infected with V-A4-cherry (red) at an m.o.i. of 5 PFU/cell for 1 h at 4°C and unbound virus was removed by washing. Non-permeabilized cells were then incubated for different times at 37°C and stained with anti-B2M to analyze colocalization. (TIF) [file ppat.1010800.s006.tif]

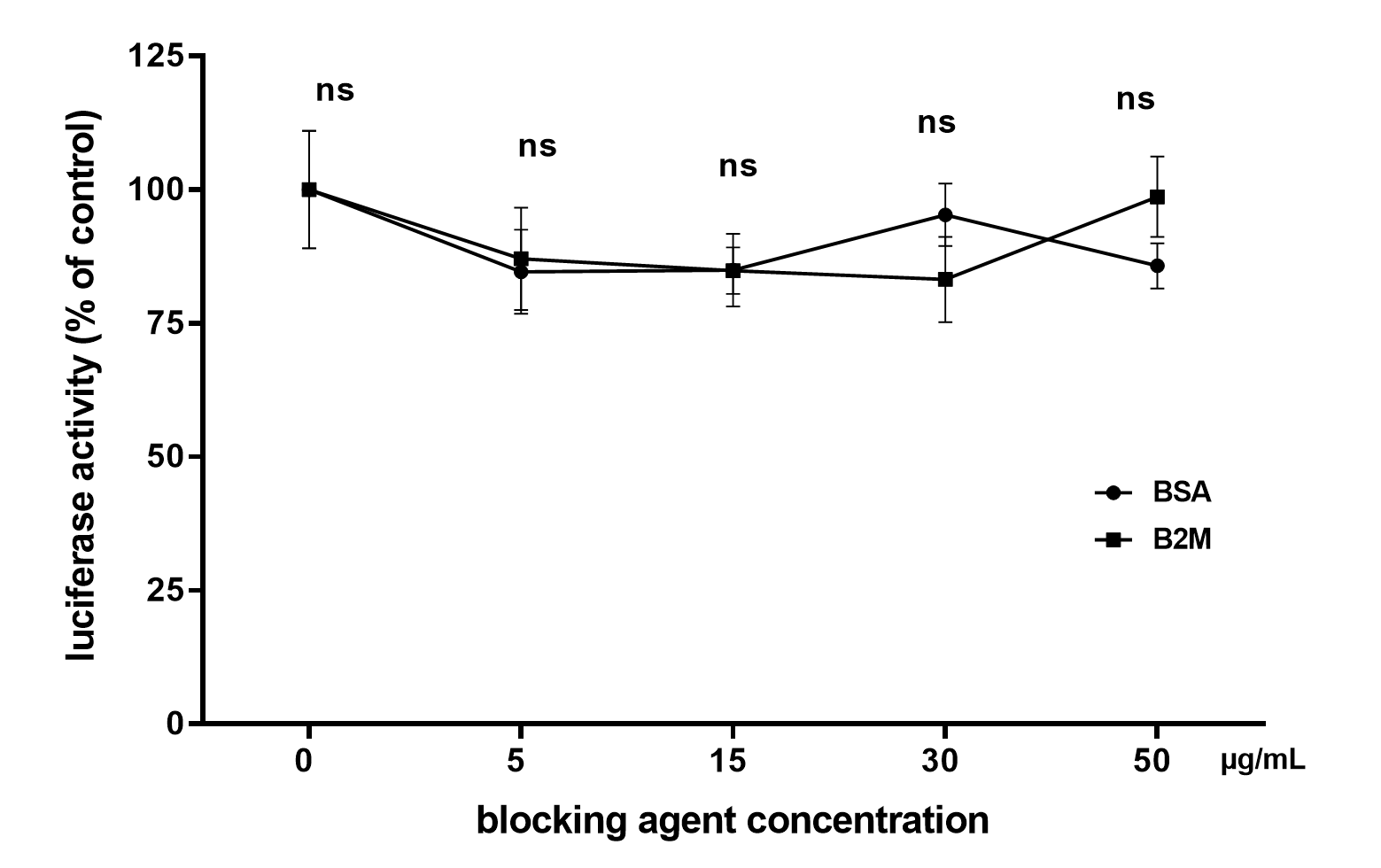

Supplement: S7 Fig — V-e.Luc virus was incubated with increasing concentrations of soluble BSA (control) or B2M (0, 5, 15, 30 and 50 μg/mL) protein for 1 h at room temperature. Then, HeLa cells were incubated for 1 h with the pre-treated inoculum. At 3 h.p.i. luciferase activity was determined as a measure of viral entry and early gene expression. No significant differences were found. p-values: **** < 0.0001, *** < 0.001, ** < 0.01, * < 0.05, ns > 0.05. (TIF) [file ppat.1010800.s007.tif]

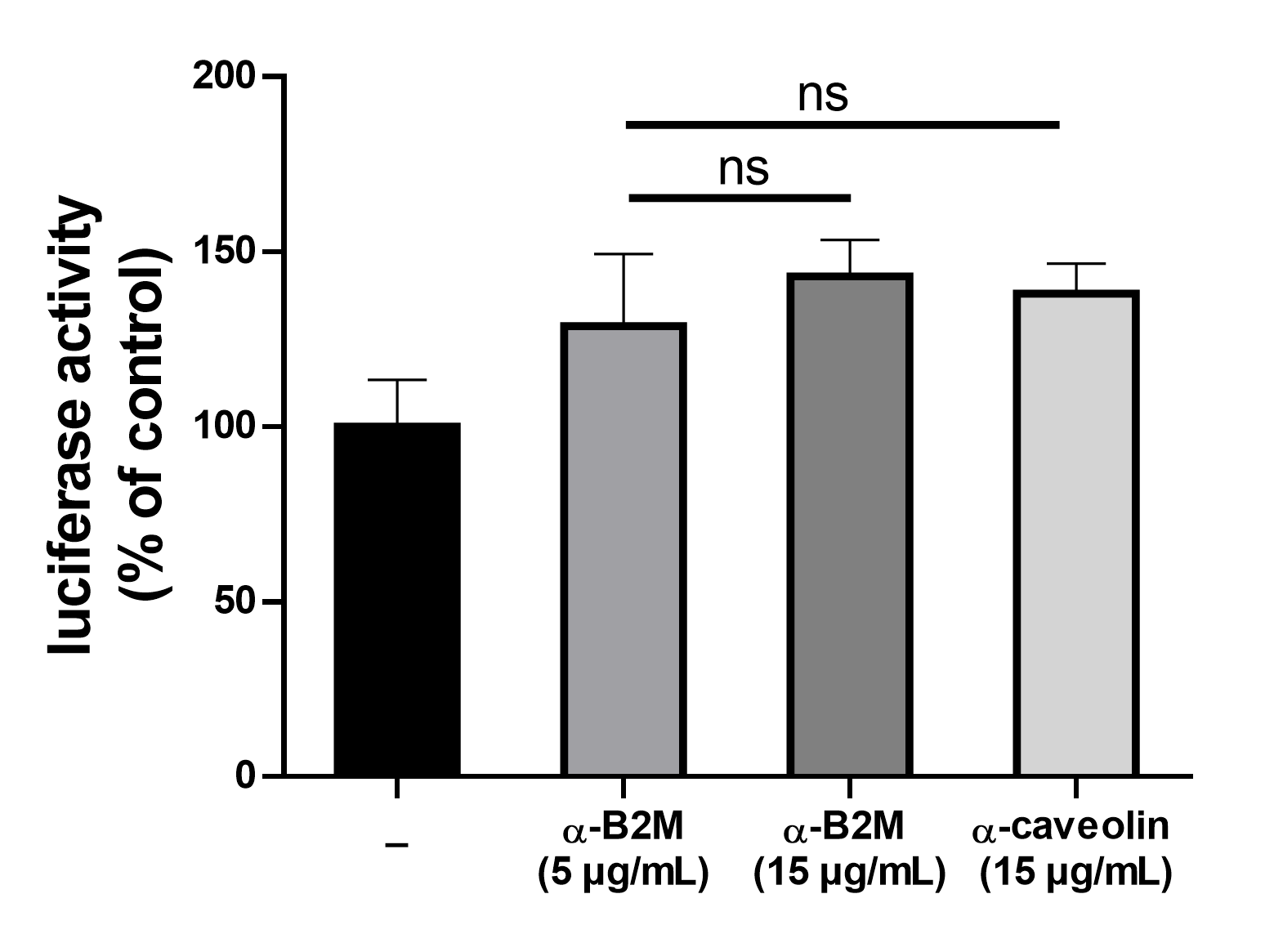

Supplement: S8 Fig — HeLa cells were incubated with two different concentrations of anti-B2M antibody (5 and 15 μg/mL) for 1 h at room temperature. Anti-caveolin antibody (15 μg/mL) was used as negative control. After antibody treatment, HeLa cells were infected with V-e.Luc (m.o.i. 0.8), and eventually 3 h.p.i. luciferase activity was determined as a measure of viral entry and early gene expression. No significant differences were found. ns, not significant (p> 0.05). (TIF) [file ppat.1010800.s008.tif]
